# Methodological issues in social cognition research in autism spectrum disorder and schizophrenia spectrum disorder: a systematic review

**DOI:** 10.1017/S0033291723001095

**Published:** 2023-06

**Authors:** Grace E. Konstantin, Julie Nordgaard, Mads Gram Henriksen

**Affiliations:** 1 Schizophrenia and Bipolar Disorder Research Program, McLean Hospital, Belmont, MA, USA; 2 Department of Psychology, The State University of New York at Binghamton, Binghamton, NY, USA; 3 Mental Health Center Amager, University Hospital of Copenhagen, Copenhagen, Denmark; 4 Department of Clinical Medicine, Faculty of Health and Medical Sciences, University of Copenhagen, Copenhagen, Denmark; 5 Center for Subjectivity Research, Department of Communication, Faculty of Humanities, University of Copenhagen, Copenhagen, Denmark

**Keywords:** autism, differential diagnosis, measurement, medication, schizophrenia, social cognition, substance use disorders

## Abstract

Recent systematic reviews and meta-analyses conclude that similar social cognitive impairments are found in autism spectrum disorder (ASD) and schizophrenia spectrum disorder (SSD). While methodological issues have been mentioned as a limitation, no study has yet explored the magnitude of methodological heterogeneity across these studies and its potential impact for their conclusion. The purpose of this study was to systematically review studies comparing social cognitive impairments in ASD and SSD with a focus on methodology. Following the PRISMA guidelines, we searched all publications on PubMed, PsycINFO, and Embase. Of the 765 studies identified in our data base searches, 21 cross-sectional studies were included in the review. We found significant methodological heterogeneity across the studies. In the 21 studies, a total of 37 different measures of social cognition were used, 25 of which were only used in 1 study. Across studies, the same measure was often said to be assessing different constructs of social cognition – a confusion that seems to reflect the ambiguous definitions of what these measures test in the studies that introduced them. Moreover, inadequate differential diagnostic assessment of ASD samples was found in 81% of the studies, and sample characteristics were markedly varied. The ASD and SSD groups were also often unmatched in terms of medication usage and substance use disorder history. Future studies must address these methodological issues before a definite conclusion can be drawn about the potential similarity of social cognitive impairments in ASD and SSD.

## Introduction

The relationship between autism and schizophrenia is long and complicated. In the beginning of the twentieth century, the concept of autism was introduced by Bleuler. Here, the concept designated detachment from reality coupled with a predominance of inner life, and it was considered a complex fundamental symptom of schizophrenia (Bleuler, 1950). On Bleuler's account, autism was not a well demarcated symptom or sign but rather a generic term, expressing a specific intersubjective displacement, which could manifest in various domains such as behavior (e.g. negativism) or cognition (e.g. idiosyncratic logic or beliefs) (Parnas, Licht, & Bovet, 2005*a*, 9). In the 1920s, Minkowski reconceived autism as the very ‘generative disorder’ of schizophrenia, defining it as loss of vital contact with reality (Minkowski, 1926), expressing a characteristic disruption of the ordinary, unmediated attunement or resonance with others and of immersion in the shared world. Other substantial studies on schizophrenic autism can be found in the works of Binswanger (1957) and Blankenburg (1971) as well as in more recent schizophrenia research (Ballerini et al., 2015; Henriksen, Raballo, & Nordgaard, 2021; Parnas et al., 2005*b*).

Through the works of Kanner and Asperger in the 1940s, the concept of autism was extracted from the psychopathology of schizophrenia and used to designate a rare syndrome with abnormalities of social relationships, stereotyped behavior, and restricted interests detectable already in infancy (Asperger, 1944; Kanner, 1943). DSM-III (American Psychiatric Association, 1980) became a crucial publication for research in what today is considered autism spectrum disorder (ASD). Here, the syndrome initially reported by Kanner and Asperger became a formal diagnosis with the introduction of the category of infantile autism. Crucially, DSM-III defined infantile autism as a *pervasive developmental disorder* and not as a kind of *psychosis* (Rutter & Schopler, 1992, 469). Previously, children exhibiting signs of this syndrome as well as other severe mental conditions had often been diagnosed with childhood schizophrenia (Rutter, 1972); a diagnostic category that was omitted in DSM-III.

In DSM-IV from 1994 (American Psychiatric Association, 1994), Asperger's disorder was introduced. Asperger's disorder shared the basic characteristics of infantile autism (which was here renamed ‘autistic disorder’) but without delays in language and cognitive development and without loss of developmental skills (American Psychiatric Association, 1994, 75.). Despite concerns about the diagnostic validity of Asperger's disorder (e.g. Ghaziuddin, Tsai, & Ghaziuddin, 1992; Rutter & Schopler, 1992; WHO: World Health Organization, 1992, 203), it quickly became a popular diagnosis. In DSM-5 from 2013 (American Psychiatric Association, 2013), the diagnostic categories of autistic disorder, Asperger's disorder, and pervasive developmental disorder were consolidated into ASD, representing a single continuum from mild to severe impairment in the domains of social interaction/communication and restrictive repetitive behaviors/interest (American Psychiatric Association, 2013, xliii). Here, the previous diagnostic onset criteria for infantile autism in DSM-III (<30 months of age) and autistic disorder in DSM-IV (<3 years of age) were diluted, requiring only symptoms to be present in the early development period, but stating that these symptoms may not be fully manifest until later in life (American Psychiatric Association, 2013, 50). Since ‘the early development period’ remains undefined and symptoms are allowed to be undetectable ‘until social demands exceed limited capacities’ (American Psychiatric Association, 2013, 50), the introduction of ASD further extended the diagnostic boundaries of autism. Correspondingly, there has been a dramatic increase in cases of autism over the last 4 decades, from 2–4 children per 10 000 in 1980 (American Psychiatric Association, 1980) to 1 in 44 children (Maenner et al., 2021).

The widening of the diagnostic boundaries of autism has enabled further overlaps with the symptomatology of other mental disorders. Today, the differential diagnosis between autism and schizophrenia, which scholars like Kanner (1943), Asperger (1944), and Rutter (1972) worked hard to establish, has again become unclear. Although ASD and schizophrenia spectrum disorders (SSD) are distinct syndromes with different clinical profiles, natural histories, and treatment options, research has emphasized points of convergence between the two syndromes, including shared genetic liability, neurobiology, psychopathology, and social cognitive impairments (Baribeau & Anagnostou, 2013; Jutla, Foss-Feig, & Veenstra-VanderWeele, 2022). Especially, overlaps in the domains of psychopathology and social cognitive impairments may have clinical implications for the differential diagnosis between ASD and SSD and subsequent treatment decisions. In contrast to studies using crude psychopathological measures, recent phenomenologically informed, empirical studies have reported crucial psychopathological differences between ASD and SSD (Nilsson et al., 2020*a*, *b*).

In this study, we focus on the reported overlap of social cognitive impairments in ASD and SSD. Systematic reviews and meta-analyses have consistently found similar social cognitive impairments in the two syndromes (Chung, Barch, & Strube, 2014; Fernandes, Cajão, Lopes, Jerónimo, & Barahona-Corrêa, 2018; Oliver et al., 2021). Nonetheless, methodological heterogeneity related to sample characteristics and test measures has been emphasized as a major limitation (Chung et al., 2014; Crespi, 2020; Oliver et al., 2021; Veddum & Bliksted, 2022). This prompts the question as to whether the claim of similar social cognitive impairments in ASD and SSD is sufficiently corroborated. Could the overlap of social cognitive impairments reflect imprecision of applied test measures to detect differences (Fernandes et al., 2018) or could it be an artifact of methodological heterogeneity across studies? Clarifying these questions may aid differential diagnostic efforts. The purpose of our systematic review is therefore to assess not the results, but the *methodology* of studies comparing social cognition in ASD and SSD. Only by assessing the studies’ methodology, can we properly assess their results and the validity of conclusions drawn across studies.

## Methods

**Figure 1.**
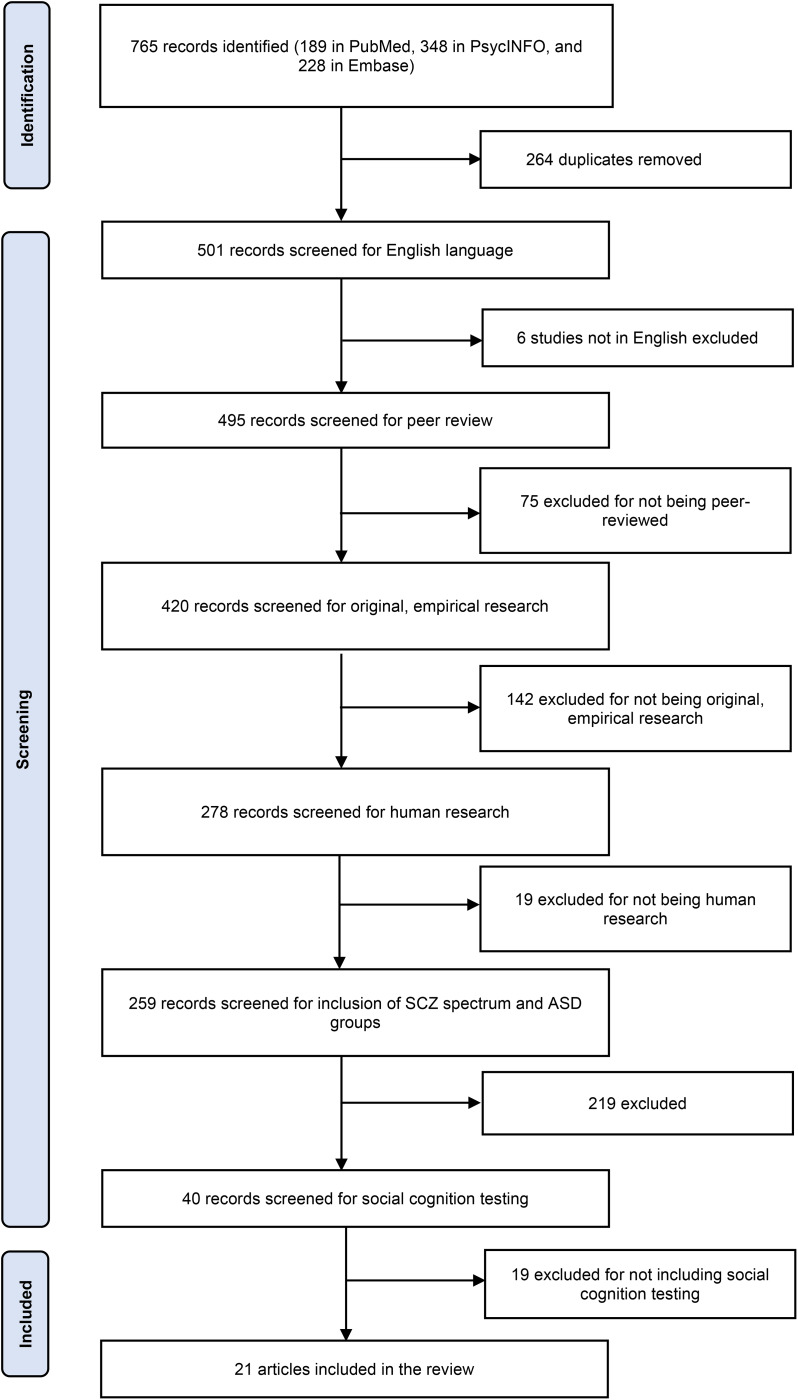
Study selection.

Following the PRISMA guidelines, we conducted a systematic review to identify studies comparing social cognition in patients with ASD and SSD. On January 20th, 2023, PubMed, PsycINFO, and Embase were searched using the following search string: schizophrenia AND autism AND ‘social cognition’. See Fig. 1 for a PRIMSA flow diagram. We applied the following inclusion criteria:

1. Studies had to be original, peer-reviewed, empirical research (not including abstracts from scientific meetings and conference proceedings)
2. Studies had to be in English
3. Studies had to be conducted on human subjects
4. Studies had to include BOTH a schizophrenia spectrum group (including schizophrenia, schizoaffective disorder, schizophreniform disorder, schizotypal personality disorder, psychosis risk syndrome, or psychosis not otherwise specified) AND an autism spectrum group (including autism, Asperger's syndrome, or pervasive developmental disorders)
5. Studies had to utilize social cognitive measures to compare the patient groups

### Data extraction

We extracted the following data from each of the eligible studies: title, authors, publication year, number of participants in each group, inclusion/exclusion criteria for each study, diagnostic assessment, age, gender, and other factors compared across groups, and methodology used to assess social cognition and neurocognition.

## Results

21 studies met our criteria and were included in the systematic review (see Table 1 for study characteristics and quality assessment). Below, we present the results of the assessment of the studies’ methodology in the following order: social cognitive measures and sample characteristics.

**Table 1.** Study characteristics

		Article	Sample (N)	Aim	Diagnostic assessments	IQ Test	QA Score^a^	Social cog. tasks used
	1	Altschuler et al., (2021)	ASD (31) SCZ (42) HC (47)	Investigate facial recognition in relation to affective ToM	DSM-IV, SCID-R, MINI, ADOS-2	WASI-II	8	BFRT, RMET
	2	Boada et al., (2020)	ASD (42) SSD: SCZ/SZA/ Schizophreniform (35) HC (70)	Compare social cognitive performance using the MASC and compare diagnostic discriminative power of MASC with other social cognition tests	DSM-IV-TR, SCID-I, ADOS-G, ADI-R, K-SADS-PL	WAIS-IV, WISC-IV, SCIP-S	6	MASC, The Strange Stories Task of Happé, The Hinting Task, The Frith-Happé Animations^b^
	3	Booules-Katri, Pedreño, Navarro, Pamias, and Obiols, (2019)	HFA (35) SSPD: Schizotypal-Schizoid Personality Disorder (30) HC (36)	Examine both cognitive and affective ToM	SCID-I and SCID-II for DSM-IV, ADI-R, ADOS	WAIS-III, WISC-IV	4	The Strange Stories Task of Happé, The Faux Pas Test, RMET
	4	Corbera et al., (2021)	HFA (30) SCZ/SZA/Psychosis Risk Syndrome (46) HC (51)	Study affective and cognitive empathy; whether empathy deficits contribute to social functioning and QOL	SCID for DSM-IV, ADOS-G	WAIS-III	8	IRI, EQ, EEPP
	5	Couture et al., (2010)	HFA (36) SCZ (44) HC (41)	Examine social cognitive abilities in SCZ and HFA	SCID-I for DSM-IV, ADI-R	WASI	5	The Point-Light Motion Displays, The Movie Stills Task, Trustworthiness Task, RMET
	6	Dubreucq et al., (2022)	ASD (30), SCZ/SZA/ Schizophreniform/ Delusional disorder/ Psychosis NOS	Compare metacognitive capacities in SSD and ASD, and associations with outcomes (i.e. social cognition)	DSM-5, AAA, ADI-R, ADOS-G	WAIS-IV, CVLT	2	MASC
	7	Eack et al., (2013)	ASD (43) SCZ/SZA (47) HC (24)	Examine neurocognitive impairment and social cognitive deficits in emotion processing in adults with ASD, SCZ and HC	SCID for DSM-IV, ADOS	CSSCEI	6	MSCEIT, Penn Emotion Recognition Test (ER-40)
	8	Graux et al., (2019)	ASD (18) SCZ/SZA (47) BP (24) HC (102)	Evaluate psychometric qualities of new French self-administered questionnaire (ACSo) assessing subjective complaints in social cognition	DSM-5, MINI, ADOS-G	Not noted	1	ACSo, STICSS, QCAU, EQ, MASC, TREF
	9	Kandalaft et al., (2012)	ASP (20) SCZ (19) HC (19)	Examine relationship between new ACS-SP test and existing social perception measures	SCID for DSM-IV, ADOS	WASI	4	ACS-SP, Ekman60, WMS-Faces, RMET (called Eyes), Triangles or Social Perception Task, MSCEIT-ME (Managing Emotion Subtest)
	10	Kuo et al., (2019)	ASD (113) SCZ/SZA (103)	Analyze whether there are relationships between MSCEIT and underlying emotion perception domains	SCID for DSM-IV, ADOS, ADI	CSSCEI, Quick Test, WASI-II	6	MSCEIT
	11	Lugnegård, Unenge Hallerbäck, Hjärthag, and Gillberg, (2013)	ASP (53) SCZ (36) HC (50)	Compare social cognitive abilities using instruments assessing different components of social cognition with low demands on verbal memory	DSM, SCID-I for DSM-IV, DISCO-11	WAIS-II Vocab Subtest	7	Animations Task^b^, RMET
	12	Martinez et al., (2017)	ASD (19) SCZ (36) HC (20)	Validate French version of the MASC, verify its discriminative value and examine correlations between social cognition,ASD and SCZ psychopathology, and age of onset	DSM-IV-TR, ADI-R, DIGS, AQ	WAIS-III	6	MASC
13		Martinez et al., (2019)	ASD: HFA or ASP (32) Recent onset SCZ (51) HC (23)	Characterize ToM impairments in relation to clinical profiles	DSM-IV-TR, ADI-R, DIGS	WAIS-III	7	Animated Shapes Task^b^
14		Morrison et al., (2017)	ASD (54) SCZ (54) HC (56)	Compare social skill using direct observation of social behaviors during a real-world situation	SCID for DSM-IV, ADOS	WRAT-3	6	SSPA
15		Nakata et al., (2020)	ASD (28) RemSZ: SCZ in Remission (28) TRS: Treatment Resistant Schizophrenia (30)	Test whether Treatment Resistant SCZ patients have more autistic traits and neurocognition/social cognition dysfunction than non-TRS	DSM-5, AQ, ASQ, PARS-TR	JART-50	9	MSCEIT, False Belief Tasks: (1) Sally-Anne's task (2) Smarties task (3) Strange Stories test (4) Sabotage/Deception task, (5) the John and Mary task
16		Pinkham et al., (2020)	ASD (101) SCZ/SZA (92) HC (101)	Compare social cognitive performance in large IQ-matched samples on a broad battery of tasks assessing different aspects of social cognition	SCID, MINI, ADOS	WRAT-3	10	AIHQ, BLERT, EmoBio, ER-40, RAD, Bio Motion, Benton Facial Recognition Task, RMET (Eyes), TASIT, The Hinting Task, CToM, The Trustworthiness Task
17		Sasson, Pinkham, Weittenhiller, Faso, and Simpson, (2016)	ASD (21) SCZ (44) HC (39)	Combine eye-tracking movement with a new task that looks at affect recognition of faces in isolation vs. in congruent/incongruent contexts	SCID for DSM-IV, ADOS	WRAT-3	6	ECT
18		Stanfield et al., (2017)	ASD (28) SPD: Schizotypal Personality Disorder (21) CM: Comorbid (10) HC (33)	Compare social cognitive deficits (on a social judgment task), and whether they are associated with different underlying activity on an fMRI	SCID-II for DSM-IV, ADOS-G	WAIS	6	Ekman60, Social Judgments Task
19		Tobe et al., (2016)	ASD (19) SCZ/SZA (92) HC (73)	Assess both visual and auditory emotion recognition to determine if unique performance profiles lend insight into shared/differing neurobiology	SCID for DSM-IV, ADOS	WAIS-III (two subtests = Processing Speed Index)	3	JL-AER, RAP, ER-40
20		Veddum, Pedersen, Landert, and Bliksted, (2019)	ASD (11) ASDHC: Healthy controls matched to ASD group (11) SCZ (21) SCZHC: Healthy controls matched to SCZ group (21)	Compare explicit and implicit ToM and social perception, as well as IQ and neurocognition	ICD-10	WAIS-IV	5	Animated Triangles Task, TASIT
21		Waris et al., (2016)	PDD: Pervasive Developmental Disorder (15) SCZ (10) SCZ/PDD (8)	Preliminary study exploring aspects of neurocognition and social cognition in SCZ, PDDs, and overlapping groups	DSM-IV, ADI-R, DISCD-11	WISC-III	6	NEPSY-II

a Quality Assessment made using a modified version of the Newcastle-Ottawa Scale.^16^ The maximum score for each study is 10 points. Low scores indicate greater risk of bias.

b Tasks are the same construction with different names.

*Note:* ACSo, Self-Assessment of Social Cognition Impairments; ACS-SP, Advanced Clinical Solutions for WAIS-IV and WMS-IV Social Perception Subset; ADI, Autism Diagnostic Interview; ADOS, Autism Diagnostic Observation Schedule; AIHQ, Ambiguous Intentions and Hostility Questionnaire; AQ, Autism-Spectrum Quotient; ASD, Autism Spectrum Disorder; ASP, Asperger's Disorder; ASQ, Autism Screening Questionnaire; BFRT, Benton Facial Recognition Test; Bio Motion, Basic Biological Motion Task; BLERT, Bell Lysaker Emotion Recognition Task; BP, Bipolar Disorder; CLVT, California Verbal Learning Test; CToM, Cartoon Theory of Mind Task; CSSCEI, Cognitive Styles and Social Cognition Eligibility Interview; DISCD, Diagnostic Interview for Social and Communication Disorders; DIGS, Diagnostic Interview for Genetic Studies; DISCO, Diagnostic Interview for Social and Communication Disorders; DSM, *Diagnostic and Statistical Manual of Mental* Disorder; ECT, Emotions in Context Task; EEPP, Empathy for Emotional Pain Paradigm; Ekman60, Facial Expressions of Emotion Stimuli and Tests; EmoBio, Emotional Biological Motion Task; EQ, The Empathy Quotient; ER-40, Penn Emotion Recognition Test; HC, Healthy Control; HFA, High Functioning Autism; ICD, *International Statistical Classification of Disease*; IRI, Interpersonal Reactivity Index; JART-50, Japanese Adult Rating Scale-50; JL-AER, Juslin & Laukka Auditory Emotion Recognition Battery; K-SADS-PL, Schedule for Affective Disorders and Schizophrenia for School-Age Children- Present and Lifetime Version; MASC, Movie for the Assessment of Social Cognition; MINI, The Mini International Neuropsychiatric Interview; MSCEIT, Mayer-Salovey-Caruso Emotional Intelligence Test; NEPSY-II, Developmental Neuropsychological Assessment; PARS, Pervasive Developmental Disorder Assessment Rating Scales; Psychosis NOS, Psychosis Not Otherwise Specified; QCAE, Questionnaire of Cognitive and Affective Empathy; RAD, Relationships Across Domains Test; RAP, Ross Attitudinal Prosody Battery; RMET, The Reading the Mind in the Eyes Test; SCID, Structured Clinical Interview for DSM; SCIP, Screen for Cognitive Impairment in Psychiatry; SCZ, Schizophrenia; SSPA, Social Skills Performance Assessment; STICSS, Subjective Scale to Investigate Cognition in Schizophrenia; SZA, Schizoaffective Disorder; TASIT, The Awareness of Social Inferences Test; ToM, Theory of Mind; TREF, The Facial Emotion Recognition Test; WAIS, Weschler Adult Intelligence Scale; WASI, The Weschler Abbreviated Scale of Intelligence; WISC, Weschler Intelligence Scale for Children; WMS-Faces, Wechsler Memory Scale: Memory for Faces Subtest; WRAT, Wide Range Achievement Test.

### Social cognitive measures

**Figure 2.**
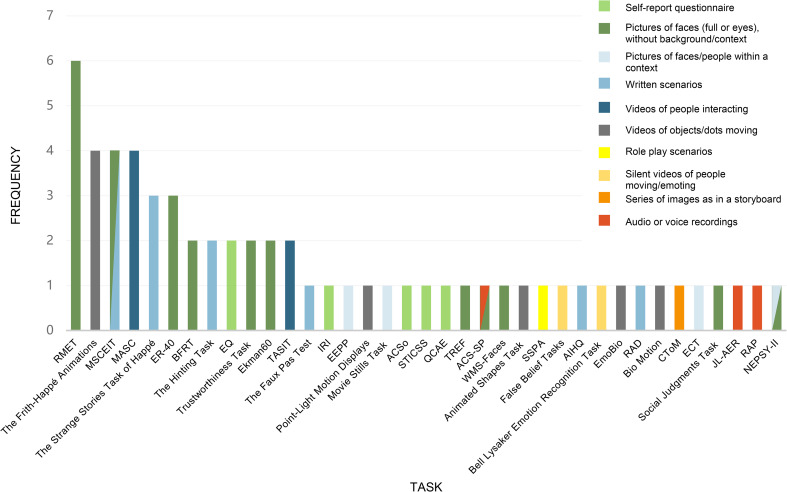
Social cognitive measures.

Across the 21 studies, 37 different measures of social cognition were used (see Fig. 2). 25 of those measures were used in only 1 study. Based on their methodology, the 37 social cognition measures can be sorted into 10 general categories: (1) self-reports or questionnaires, (2) tasks requiring participants to view still images of faces or eyes without any background or context, (3) tasks involving still images of people within a context, (4) tasks requiring participants to read written social scenarios and answer questions, (5) tasks involving watching videos of people interacting and conversing, (6) tasks including videos of people moving and emoting in silence, (7) tasks involving watching videos of objects, shapes, or dots moving, (8) tasks involving in-person role play with an experimenter, (9) tasks which had participants view a series of images with text, as in a storyboard, (10) tasks involving listening to audio or voice recordings (see Fig. 2).

For example, the most frequently used measure was the Reading the Mind in the Eyes Test (RMET) (Baron-Cohen, Wheelwright, Hill, Raste, & Plumb, 2001) also referred to as ‘Eyes’ or ‘the Eyes Task’. This task was utilized in six studies and requires participants to recognize emotions and mental states in photographs of the eye region of different faces and choose the most accurate descriptor for the thought or feeling being portrayed. Unlike many social cognition tasks, this task does not provide any situational details or context for the emotion states. The Frith-Happé Animations, also referred to as ‘Triangles’ or the ‘Social Perception Task’ (Abell, Happé, & Frith, 2000), were used in four studies. Here, participants are asked to watch a series of short, animated clips of triangles with varying patterns of movement and then classify the movement in the clip as random, goal-directed, or implying a mental state attribution. The Mayer-Salovey-Caruso Emotional Intelligence Test (MSCEIT), which is a subtest within the MATRICS Consensus Cognitive Battery (Green et al., 2004), also appeared in four studies. It is primarily comprised of written stories of emotional problems and the participants are asked to answer questions about consequences of one's thoughts, feelings, and actions.

Tasks that were used in several studies were, across studies, often described as testing different social cognitive constructs. For example, the RMET was said to assess for emotion recognition, facial affect recognition, affective Theory of Mind (ToM), social perceptual ToM, social perception, or mental state attribution; The Frith-Happé Animations was said to assess for ToM, implicit ToM, or mental state attribution; The MSCEIT was said to assess for emotion processing, emotional intelligence, emotional perception, or understanding and modulation of emotions; and The Movie for the Assessment of Social Cognition (MASC) was said to assess mental states, over and under mentalizing, or ToM (see Table 2 for descriptions of what the measures that were used in >2 studies were said to assess and what these measures were said to assess by their developers).

**Table 2.** Descriptions of social cognition constructs utilized in >2 studies

Social cognition measure	Number of studies utilizing this measure	Constructs this measure is said to assess across review studies	Original descriptions of measurement
Reading the Mind in the Eyes (RMET)	6	Emotion recognition^1,4^, facial affect recognition^5^ affective Theory of Mind (ToM)^1^, social perceptual ToM^3^, mental state attribution^2,6^, or social perception^5^	“…we described it as an “advanced theory of mind test”.” “Theory of mind is also referred to as “mentalising” (Frith, Morton & Leslie, 1991), “mind reading” (Whiten, 1991), and “social intelligence” (Baron-Cohen, Jolliffe, et al., 1997), and overlaps with the term “empathy”.” ‘We had succeeded in developing a test of social sensitivity or mind-reading that was able to reveal subtle mind-reading difficulties in adults with HFA or AS.’ (Baron-Cohen et al., 2001)
Frith-Happé Animations	4	ToM^4,5,7^, implicit ToM^8^, or mental state attribution^8^	‘This aspect of social intelligence has been referred to as ‘theory of mind’ (ToM) or ‘mentalising’’ ‘The aim of this study was to design novel stimuli that would selectively evoke mental state attribution by their motion properties.’ (Abell et al., 2000)
Mayer-Salovey-Caruso Emotional Intelligence Test (MSCEIT)	4	Emotion processing^9^, emotional intelligence^9,10^, emotional perception^11^, or the understanding and modulation of emotions^4^	‘The MSCEIT is intended to measure four branches, or skill groups, of EI: (a) perceiving emotion accurately, (b) using emotion to facilitate thought, (c) understanding emotion, and (d) managing emotion’ ‘A new ability test of EI (emotional intelligence)’ (Mayer, Salovey, Caruso, & Sitarenios, 2003)
Movie for the Assessment of Social Cognition (MASC)	4	Over and under-mentalizing^7,12^, mental states^13^, or ToM^7,12,14^	‘The ability to attribute mental states to oneself and others is referred to as social cognition or theory of mind.’ ‘The present report describes the development of another (…) instrument for the assessment of social cognition. The Movie for the Assessment of Social Cognition (MASC) requires study subjects to make inferences about video characters’ mental states’ ‘In the present study we introduced the MASC, a new tool for the assessment of mindreading abilities in individuals with a diagnosis of Asperger syndrome.’ (Dziobek et al., 2006)
Strange Stories Task of Happé	3	ToM^11^, cognitive ToM^2^, False belief^11^, ‘inference about thoughts, emotions, intentions’^7^	‘The aim was to extend the range of tasks involving theory of mind to a more contextually embedded and realistic form, which might be expected to trip up even those subjects who succeeded on the previous, simplified tasks’ (Happé, 1994)
Penn Emotion Recognition Test (ER-40)	3	Emotion recognition^6,9,15^	‘The authors used color photographs of emotional and neutral expressions to investigate recognition patterns of five universal emotions in schizophrenia’ (Kohler et al., 2003)

1 Altschuler et al. (2021), ^2^Booules-Katri et al. (2019); ^3^Couture et al. (2010); ^4^Kandalaft et al. (2012); ^5^Lugnegård et al. (2013); ^6^Pinkham et al. (2020); ^7^Boada et al. (2020); ^8^Veddum et al. (2019); ^9^Eack et al. (2013); ^10^Kuo et al. (2019); ^11^Nakata et al. (2020); ^12^Graux et al. (2019); ^13^Martinez et al. (2017); ^14^Dubreucq et al. (2022); ^15^Tobe et al. (2016).

### Sample characteristics

The 21 studies included a total of 1733 patients: 779 with ASD and 954 with SSD. Across studies, the weighted mean age was 25.2 for ASD and 30.5 for SSD.

#### Diagnostic makeup

In 14 studies, the ASD sample was defined precisely as ASD. In 6 studies, the ASD sample consisted only of patients with high-functioning autism (HFA) or Asperger's disorder, and 1 study the sample consisted of patients with pervasive developmental disorder. In 10 studies, the SSD sample consisted only of patients with schizophrenia, and in 5 studies the SSD sample included patients with schizophrenia or schizoaffective disorder. In the remaining 6 studies, the SSD sample was slightly different (see Table 1).

To diagnose ASD, 16 studies used AAA, ADOS, ADI, or DISCO, 2 studies used ADOS for some but not all patients with ASD, and 3 studies did not specify the diagnostic method. To diagnose SSD, 14 studies used SCID/SCID-II, 2 studies used DIGS, and 5 studies did not specify the diagnostic method. Only 4 studies conducted a sufficient differential diagnostic assessment of both their SSD and the ASD group (Altschuler et al., 2021; Boada et al., 2020; Martinez et al., 2017; Martinez et al., 2019). The remaining 17 studies (81%) used apparently solely an insufficient, specialized diagnostic method (AAA, ADOS, ADI, or DISCO) to diagnose ASD, meaning these 17 studies did not conduct a comprehensive differential diagnostic assessment of this group. If such comprehensive assessments were, in fact, conducted in these studies, it has not been transparently conveyed in the published articles.

#### IQ

18 of the 21 studies reported an estimated IQ, utilizing varying versions of the WASI, WAIS, WISC, WRAT-3, SCIP, Jart-50, and Quick Test. Each of these studies reported a mean IQ for each diagnostic group, except for Graux et al. (2019), which only reported an average IQ for their ASD group. Dubreucq et al. (2022) reported WAIS-IV short-term memory and working memory subtest scores only. 16 of the 18 studies reporting IQ, reported IQ averages above 100 for their ASD group. Across the studies, the IQ weighted average was 105.31 (s.d. = 7.75) for the ASD group and 100.22 (s.d. = 6.69) for the SSD group, respectively.

#### Medications

Across the 21 included studies, 13 studies reported participants’ medication usage in some fashion, while 8 studies did not. Of the 13 studies that recorded medication, 4 of them reported that every participant in the SSD group received at least 1 antipsychotic medication, while the ASD group was not on any medication. In each of the remaining 9 studies, the SSD group was more frequently on antipsychotics, more frequently on combinations of multiple antipsychotics, and prescribed higher dosages than their ASD counterparts. In 8 studies, it was reported that some of the participants in the ASD group were prescribed antipsychotics. 7 studies had exclusion criteria related to medication usage, e.g. not allowing for changes in medications within a certain time-period or for antipsychotic dosages above a certain chlorpromazine equivalent threshold. 2 studies (Eack et al., 2013; Kuo, Wojtalik, Mesholam-Gately, Keshavan, & Eack, 2019) required that the SSD group received antipsychotic medication.

#### Substance use

2 of the 21 included studies reported participants’ history of substance use disorder. In Kuo et al. (2019), substance use disorder was noted only for the SSD group, revealing that 44% of SSD participants had substance use disorder. In Eack et al. (2013), 60% of participants in the SSD group met criteria for substance use disorder. In both studies, it was unclear if these instances of substance use disorder were current or lifetime.

## Discussion

In this review, we investigated the *methodology* of studies comparing social cognition in SSD and ASD. Upon reviewing the literature, serious methodological issues became evident, which collectively question the validity of the main result from recent systematic reviews and meta-analyses, namely that of similar social cognitive impairments in ASD and SSD. In sum, we found that the measures used to assess social cognition were remarkably heterogenous, there was little consensus about what domains of social cognition the many measures actually assessed, and there were methodological issues pertaining to diagnostic assessment and sample characteristics. Below, we discuss each of these issues in turn.

We identified 37 different measures of social cognition used across the 21 reviewed studies, with 25 measures appearing in only a single study each. These tasks vary greatly in how they are constructed and administered, and they range from identifying elements of photographs to watching shapes move in a video to reading and responding to written social scenarios. This diversity testifies to a pervasive heterogeneity in the methodology for assessing social cognition. It also emphasizes that ecological validity remains a substantial issue for most of these measures (Beer & Ochsner, 2006; Revsbech et al., 2017). Put differently, *reflecting* upon and forming judgements about the movement of shapes or dots, emotions expressed in the eye region only, or what takes place in videos or written scenarios seem far removed from real-life, contextual social *interactions*. Real-world social interactions take place on a backdrop of a basic, immediate attunement between the interacting individuals. The social cognitive measures do not tap into this basic level of interpersonal attunement, which according to both founding and contemporary scholars in schizophrenia and autism research is where the root problems, different as they may be, lie in these disorders (Asperger, 1944; Blankenburg, 1971; Bleuler, 1950; Hobson, Chidambi, Lee, & Meyer, 2006; Kanner, 1943; Minkowski, 1926).

Another issue is the conceptual ambiguity surrounding the definitions of the domains of social cognition and how these domains were tested. The same measure – administered in the same way – was often used to test different domains of social cognition across the different studies. For example, the RMET was said to assess emotion recognition, affective ToM, social perceptual ToM, or mental state attribution depending on the study. Notably, this conceptual confusion is not really a matter of the authors of the reviewed studies mislabeling the targeted social cognitive domains of the measures they use. Rather, the confusion seems mainly to stem from ambiguous and imprecise definitions of what these measures test in the original studies that introduced them (see Table 2). To illustrate some of these basic problems, we here focus on the most used measure, the RMET.

In the study that introduced the RMET (Baron-Cohen et al., 2001), it is described as an ‘advanced theory of mind test’. Referencing Premack and Woodruffs’ (1978) classical study on ToM in chimpanzees, ToM is defined as ‘the ability to attribute mental states to oneself or another person’ (Baron-Cohen et al., 2001). The authors state that ToM ‘is the main way in which we make sense of or predict another person's behaviour’; that ToM is also referred to as ‘mentalizing’, ‘mind reading’, ‘social intelligence’; that ToM ‘overlaps’ with ‘empathy’ (cf. Premack & Woodruff, 1978, 518); and that RMET measures ‘social sensitivity or mind-reading’. Several basic problems can be pointed out: (1) The abundance of partially overlapping but clearly not identical concepts induce confusion about what the RMET examines from the very outset. This confusion could be solved by specifying each of these concept's extension (i.e. the set of objects to which it applies) and intension (i.e. the properties connected to it) but no such attempt is made in the study. (2) The authors are apparently unsure about whether their measure tests ‘social sensitivity *or* mindreading’ (our emphasis). (3) ToM is a broad construct, concerning our ability to ascribe mental states like intentions, beliefs, knowledge, and emotions to others, and the guiding assumptions are that (i) these ascriptions are based on inferences and (ii) that we make inferences because others’ mental states are not directly observable to us. Given that ToM is such a broad construct, it seems questionable, at least, that the RMET, which narrowly tests emotion recognition in still photos of the eyes can be said to test ToM as such. Put differently, does performance on the RMET enable us to draw conclusions about the person's capacities for ToM, social sensitivity, or social intelligence beyond the specific tasks of emotion recognition examined in RMET? Is it not imaginable that a person may perform poorly on the RMET and still be able to attribute mental states like intentions, beliefs, knowledge, or emotions to others?

We fully recognize that carving out and delimiting domains of social cognition for specific measures is not an easy task. Yet, the conceptual confusion surrounding the definition of the original measures is telling for the variety of labels of social cognitive domains these measures subsequently have been said to test. If we do not have a firm conceptual grasp of the constructs or phenomena we aim to study and assess, our empirical research is not likely to yield clear results (Marková & Berrios, 2016). When the delineation between domains of social cognitions is so blurred and the same measure is said to be assessing different domains, it becomes difficult to draw any solid conclusion about the character of the social cognitive impairments being measured and about the shared *vs*. distinct nature of social cognitive impairments in ASD and SSD, though such distinctions could provide important targets for etiological research.

To advance research on social cognition, interdisciplinary collaboration, combining theoretical models of social cognition, which conceptually carve out its inner domains and their boundaries, and empirical studies, testing the discriminative power of the different measures in accordance with these domains is strongly needed. While testing the psychometric properties of different social cognitive measures is crucial to this end (see below), it is of utmost importance to conceptually delineate the social cognitive domains these measures test – good psychometric properties cannot compensate for lack of conceptual delineation of what the measure tests. Paraphrasing an insight by Kendler (1990), psychiatric research is confronted by both empirical and ‘nonempirical’ issues (e.g. the conceptual clarity of the constructs or phenomena we study empirically) and they both need to be considered for psychiatric research to thrive and prosper.

The abundance of measures used testifies to the importance of research like The Social Cognition Psychometric Evaluation (SCOPE) study (Pinkham et al., 2014; Pinkham, Harvey, & Penn, 2018), which assesses the psychometric validity of social cognitive measures. One of the findings from SCOPE was that RMET – the most frequently used social cognitive measure across the included studies in our review – did not show sufficient psychometric properties to be evaluated as ‘acceptable’. By contrast, the 3 measures, which in the SCOPE study were evaluated as ‘acceptable’ and recommended for use in clinical trials, were only used in 4 of the 21 included studies in our review: The Penn Emotion Recognition Test (ER-40) was used in 3 studies (Eack et al., 2013; Pinkham et al., 2020; Tobe et al., 2016), The Hinting Task in 2 studies (Boada et al., 2020; Pinkham et al., 2020), and The Bell Lysaker Emotion Recognition Task (BLERT) in 1 study (Pinkham et al., 2020). Prioritizing measures with the best psychometric properties will solve many problems related to test heterogeneity.

As noted briefly above, ecological validity is also an issue in many of the used measures and it deserves some unpacking in this context. The construct of ecological validity is usually divided into ‘veridicality’, referring to the degree to which a measure correlates with measures of real-life functioning, and ‘verisimilitude’, referring to the degree to which the cognitive demands of a measure resemble the cognitive demands at stake in real-life situations (Chaytor & Schmitter-Edgecombe, 2003; Franzen & Wilhelm, 1996). In the SCOPE study (Pinkham et al., 2018), ecological validity of the social cognitive measures was assessed to some extent in terms of ‘veridicality’, finding some correlations between these measures and functional outcome in schizophrenia.

The other aspect of ecological validity, ‘verisimilitude’, is perhaps even more challenging. Admittedly, it may be very difficult to design a measure of social cognition that has perfect verisimilitude, because every test situation of social cognition might be a somewhat artificial setup compared to real-life social cognition. In principle, however, it is possible to differentiate between degrees of verisimilitude by providing arguments for which *methodologies* of the social cognition measures that best approximate real-life social cognition – e.g. should priority be given to measures that target humans (instead of moving shapes or dots), measures that include situational or contextual information, and/or to measures that entail interactional elements to better resemble real-life social cognition? In the Results section ‘Social cognitive measures’, we sorted the 37 applied measures of social cognition into 10 different categories based on their methodology. This division may serve as a preliminary reference for reflecting upon and providing arguments for assessing the verisimilitude of these measures. While there is a need for future research to develop new social cognitive measures with a high degree of verisimilitude, the success of such new measures hinges on the described interdisciplinary work of conceptually carving out the inner domains of social cognition and delineating their boundaries.

Regarding sample characteristics, we found several critical issues. First, it is of major concern that 17 studies (81%) apparently relied solely on an insufficient, specialized diagnostic method to assess ASD. Without conducting a comprehensive differential diagnostic assessment, we cannot be sure that the patients with ASD in these studies are correctly diagnosed. Although they fulfill diagnostic criteria for ASD, they may also fulfill criteria for other mental disorders, including SSD. Although some studies state that they excluded patients with ASD with a comorbid diagnosis of SSD or a psychotic disorder, these disorders cannot be ruled out when the patients with ASD were not assessed for such disorders. Given overlaps between ASD and SSD (Jutla et al., 2022), this is a crucial issue. For example, a recent nationwide cohort study of 11 170 adolescents and adults with ASD found a progression rate to schizophrenia of 10.26% (Hsu et al., 2022; Lugo Marín et al., 2018). To tackle this issue, future studies must conduct comprehensive differential diagnostic assessment of their sample, including their ASD groups.

Another recurring issue was attempts to draw conclusions from samples that were not adequately matched – e.g. comparing HFA (which only represents a part of ASD) to chronic schizophrenia (which also only represents a part of SSD). This issue was also reflected in the IQ assessments. Of the 21 included studies, 16 reported IQ averages of >100 for their ASD sample. This indicates that not many patients in the more severe end of ASD were included in the sample. For example, a recent birth cohort study found that in the group with the most inclusive definition of ASD, 59.1% had an IQ score in the range of average or higher (average defined as 86 to116), meaning an estimated 40.9% of participants should have an IQ score of 85 or below (Katusic, Myers, Weaver, & Voigt, 2021).

Another issue related to sample matching is medication usage, which was often not reported at all. In studies that did report it, the samples drastically differed in medication usage both within and across studies. In more than half of the studies, medication usage was noted in some fashion, but not always controlled for. In four studies, all patients in the SSD group were taking at least one antipsychotic, while the ASD sample were taking none. Medication usage is an important issue to consider because psychotropic medication has been shown to affect general cognition as well as social cognition – e.g. a recent meta-analysis (Oliver et al., 2021) found that as antipsychotic treatment increased, ToM performance decreased. We agree with the authors of this meta-analysis, who argue that future studies must assess how antipsychotic treatment affects social cognition across ASD and SSD.

A final issue about group matching concerns substance use. Most studies did not record substance use, and in the two studies that did, it was unclear whether patients had current and/or lifetime substance use disorders. In these studies, only patients with SSD had some sort of substance use disorder. Since current and historic substance use disorders may impact cognitive performance (Bora & Zorlu, 2017; Potvin et al., 2018), the issue of substance use must also be addressed in full detail in future studies.

In our view, the methodological issues discussed above collectively indicate a more global need for a renewed focus on methodological rigor in psychiatric research. Without a solid methodological basis, the validity, applicability, and clinical relevance of empirical results remain dubious. Perhaps with the intention of solving some of these issues, a general trend in contemporary psychiatric research, also found in our review, is to create ever new tests or scales and validate them against existing ones. In our view, such new tests or scales rarely contribute to advance psychiatric knowledge but instead they unintentionally end up further increasing methodological heterogeneity as was the case in our study.

## Conclusion

We found substantial and pervasive methodological heterogeneity across studies, which collectively questions the validity of the reported finding of similar social cognitive impairments in ASD and SSD. Drawing this conclusion seems premature. By highlighting shortcomings in the contemporary literature, we have emphasized challenges and possible solutions for future research on social cognition in clinical populations. Specifically, we emphasize a need for (i) interdisciplinary efforts to improve delineation of social cognitive domains and identify suitable measures for each domain, (ii) increased homogeneity in measures used to assess social cognition, and (iii) improving differential diagnostic assessment and group matching.

## Authors’ contributions

The authors jointly identified the study's objective, search string, and selection criteria for the systematic review. GEK searched the data bases and all authors participated in the sorting of articles, data extraction, and quality assessment. All authors discussed the study's results and their interpretation. GEK wrote a first draft of the manuscript, which was substantially revised by MGH and JN. All authors approved the final version.
